# Adverse Reactions to Radioiodine 131I Therapy of Goiter in West African Tertiary Hospital

**DOI:** 10.4274/mirt.13007

**Published:** 2016-09-29

**Authors:** Yetunde A. Onimode, John E. Ejeh, Akintunde T. Orunmuyi

**Affiliations:** 1 University of Ibadan College of Medicine, Department of Radiation Oncology, Nuclear Medicine Unit, Ibadan, Nigeria; 2 University College Hospital, Department of Nuclear Medicine, Ibadan, Nigeria

**Keywords:** Adverse reactions, Radiotherapy, Thyroid neoplasms, nuclear medicine

## Abstract

**Objective:**

Radioactive iodine therapy (RAIT) is established as an efficient means of treating toxic goiter (TG) globally. The field of nuclear medicine (NM) still appears novel to many Nigerian clinicians and patients. A culturally embedded dread of radiation may raise ethical and moral concerns about potential adverse effects in the wake of RAIT in our setting. An adverse drug reaction may be described as “a response to a drug which is noxious and unintended, and which occurs at doses normally used in man”. This study therefore, seeks to review adverse reactions (ARs) experienced following RAIT. We would also like to improve patient and physician education about the safety profile of RAIT.

**Methods:**

This is a retrospective analysis of all patients who had received RAIT for thyroid disease from August 2006 to June 2015.

**Results:**

Forty typical ARs were experienced following 36 therapy sessions (18.65%) with RAIT in 35 patients (21.47%) aged 17-78 years, of which three had multiple sessions for well-differentiated thyroid carcinoma (WDTC).

**Conclusion:**

RAIT remains a safe option for the treatment of benign and TG. The experienced ARs are mainly mild to moderate in severity and mostly short-lived. As larger doses of radioactive iodine for WDTC and TG were more commonly associated with ARs, our study suggests that these patients merit stronger prophylactic measures as well as closer monitoring for earlier detection and management of these reactions.

## Abstract (tr)

**Amaç:**

Radyoaktif iyot tedavisi (RAIT) tüm dünyada toksik guatr (TG) tedavisinde etkin bir araç olarak kullanılmaktadır. Nükleer tıp (NM) alanı hala birçok Nijeryalı klinisyen ve hasta için yeni kabul edilmektedir. Radyasyondan kültürel bir korku RAIT kullanımında olası yan etkilerle ilgili etik ve ahlaki kaygıları arttırabilir. Bir ilaç yan etkisi “bir ilaca karşı normal insanlarda kullanılan dozlarda oluşan zararlı ve istenmeyen tepki” olarak tarif edilebilir. Bu çalışma bu nedenle RAIT’ye bağlı yan etkileri gözden geçirmeyi amaçlamıştır. Aynı zamanda RAIT güvenlik profili hakkında hastaları ve hekimleri eğitmeyi de arzu ediyoruz.

**Yöntem:**

Bu çalışma Ağustos 2006-Haziran 2015 tarihleri arasında tiroid hastalığı için RAIT almış tüm hastaların retrospektif bir analizidir.

**Bulgular:**

Yaşları 17-78 arasında 35 RAIT (%21,47) hastasının 36 (%18,65) tedavi seansında kırk yan etki gözlenmiştir, üç hastaya iyi diferansiye tiroid karsinomu nedeniyle çoklu seans uygulanmıştır.

**Sonuç:**

RAIT iyi huylu ve TG tedavisi için güvenli bir seçenek olmaya devam etmektedir. Gözlenen yan etkiler genellikle hafif-orta şiddette ve çoğunlukla kısa ömürlüdür. İyi diferansiye tiroid kanseri ve TG için uygulanan yüksek dozlar daha çok yan etki ile ilişkili olduğundan çalışmamız bu hastalarda daha güçlü profilaktik tedbirlerin yanı sıra bu reaksiyonların erken saptanması ve yönetimi için daha yakından izlenmelerini önermektedir.

## INTRODUCTION

^131^I radioactive iodine (RAI) is one of the 15 known radioisotopes of iodine, and is the most widely used in the diagnosis and therapy of thyroid diseases. RAI is reactor produced from the fission of ^235^U (1,2). RAI is one of the most widely used radioisotopes in nuclear medicine (NM), the most popular being ^99m^Tc. RAI, like stable iodine, is trapped and organified in the thyroid gland. It emits two types of radiation: 364 and 664 keV gamma rays (for imaging) and 192 keV beta particles (for therapy), respectively (3). Its beta particles deliver a lethal radiation dose to the thyroid cells that accumulate them.

Radioactive iodine therapy (RAIT) has been in use for the treatment of thyroid diseases for more than seven decades (4). It has been established as an efficient means of treating toxic goiter (TG) globally. Indications for RAIT include well-differentiated thyroid carcinoma (WDTC), primary hyperthyroidism due to Graves’ disease (GD), toxic multinodular goiter and toxic adenomas, and for thyroid size reduction in cases of sporadic non-toxic/euthyroid goiter (EUG). There has been an increase in the use of RAIT as first line therapy for GD and the treatment of choice for recurrent GD and toxic nodular hyperthyroidism (5,6).

RAIT has recently been introduced to the management of patients with benign and malignant thyroid disease in Nigeria. The field of NM still appears novel to many Nigerian clinicians and patients (7,8). Unfamiliarity of clinicians with the efficacy of this modality has been encountered and its consequence on patient referral is unknown. Earlier studies in our environment reported acceptable treatment response rates of 83.87% and 77.3%, respectively, confirming the efficacy of RAIT for hyperthyroidism (9,10). A culturally embedded dread of radiation may raise ethical and moral concerns about potential adverse effects in the wake of RAIT in our setting such as infertility (11,12).

An adverse drug reaction may be defined as “an appreciably harmful or unpleasant reaction, resulting from an intervention related to the use of a medicinal product, which predicts hazard from future administration and warrants prevention or specific treatment, or alteration of the dosage regimen, or withdrawal of the product” (13). It has also been described as “a response to a drug which is noxious and unintended, and which occurs at doses normally used in man” (14,15). Unlike radiotracers, RAI has ARs related to its associated radioactivity and not due to an “unanticipated physiologic response to the vehicle (tracer) carrying the radioactivity” (16). The previously reported frequency of ARs to radiopharmaceuticals are 11/100.000 in Europe, 2.3/100.000 in the US, and more recently, 0.8/100.000 in Japan (17,18,19). The figure quoted for the US remains relatively unchanged from the earlier frequency of 2.3/100.000 (20). The prevalence of ARs in NM is approximately 1000­fold less than that quoted for iodinated contrast media and drugs; these are as high as 19.4% (21,22). Side effects of the treatment of goiter are known to negatively impact patient care (Table 1).

This study therefore, seeks to retrospectively review ARs experienced following RAIT. We would also like to improve patient and physician education about the safety profile of RAIT. This would further enhance patient care and safety in relation to RAIT. To the best of our knowledge, this is the first West African study to address this issue.

## MATERIALS AND METHODS

This is a retrospective analysis of all patients who had received RAIT for thyroid disease from August 2006-June 2015. Patients were treated based on empirical estimates for benign goiter [(TG) and EUG] as well as WDTC. Their management protocols are as follows:

### General measures:

 RAIT is given as empirical doses; its capsules have been initially ordered from Amersham, South Africa, but they were ordered from IBA Molecular, France since February 2011. All patients fasted for at least two hours prior to RAIT, and two hours afterwards. At radioactive doses less than or equal to 555 MBq, patients were treated on an outpatient basis and discharged home after having been observed for possible ARs.

### On admission:

 Patients who received radioactivity exceeding 555 MBq were admitted to our isolation wards, typically those with EUG and WDTC. Patients with TG who received doses exceeding 555 MBq of ^131^I also followed this protocol. Prophylactic measures against ARs were taken; the prescription of pain relievers (paracetamol or non-steroidal anti-inflammatory drugs unless contraindicated), mist magnesium trisilicate or other antacid, lime juice or chewing gum for salivary gland protection, as well as liberal oral fluid intake as tolerated. Admitted patients were monitored daily and patients were discharged home at radiation dose readings ≤555 MBq at one meter from the patient. From August 2012, metoclopramide prophylaxis has been strictly enforced to prevent vomiting.

Follow-up clinic visits were scheduled at one month post-RAIT for all patients in order to assess their clinical status and to assess their hematological profiles for possible cytocidal effects of radiation. Patients were asked to report any ARs experienced following treatment during their admission and at follow-up sessions.

In addition, statistical analysis of patients who had received radioiodine therapy was performed using IBM statistics SPSS software version 21. The chi-square test was performed to test for significant association between presence or absence of adverse effects according to patient age groups (less than or equal to 44 years, or greater than 44 years), gender, type of diagnosis (TG, EUG, WDTC) and malignancy of goiter (benign or malignant), RAI treatment (less than or equal to 64 mCi-being the upper dose limit for benign goiters, or more than 64 mCi).

Nausea and vomiting, being the most common AR, was also tested for significant association with the above factors, as well as presence or absence of antiemetic therapy pre-RAI.

## RESULTS

Records were available for a total of 193 RAI treatments administered to 163 patients between 23 August, 2006 and June 8, 2015. Patient characteristics are presented in Table 2.

Forty typical ARs were experienced following 36 therapy sessions (18.65%) with RAIT in 35 patients (21.47%) aged 17-78 years, of which three had multiple sessions for WDTC (Table 3). All observed ARs were classified as early, relative to the period of occurrence post RAIT, and were also grouped as being mild to moderate in severity (67.5% mild, 32.5% moderate) (Table 4) (23). There were no mortalities. ARs were most common in WDTC (27 reactions; 67.5%), less so with TG (nine reactions; 22.5%) and the least in those with EUG (four reactions; 10%). The overall frequency of ARs in all NM procedures, whether diagnostic or therapeutic, performed during the study period was 0.78%.

A female preponderance was noted in reported ARs (male: female ratio of 1:5). This is likely subsequent to the pre-existing bias in the patient population.

Regarding goiter size, the greater the quantity of residual thyroid tissue the greater was the frequency of ARs observed (13 had no prior thyroid surgery, 12 had subtotal or near-total thyroidectomies, three had total thyroidectomies, two had lobectomies, while the nature of surgery was not known for five). Of the 12 patients who had been operated upon, the intense “star artifact” was seen in four patients in the thyroid bed on ^131^I scanning, implying significant residual functioning thyroid tissue. In an additional seven, both thyroid lobes were visualized. However, the extent of thyroid visualization despite thyroidectomy varied depending on the operating surgeons. Two patients who had been operated upon subsequently developed metastatic disease prior to RAIT.

RAI therapy patients with doses of ^131^I less than or equal to 64 mCi were significantly less likely to experience ARs than those treated with higher doses; p=0.042. Also, patients with benign goiter (TG or EUG) were less prone to have ARs as compared to those with malignant goiters (WDTC); however, this was not significant; p=0.06. Despite the preponderance of female patients having ARs, gender proved non-significant; p=0.11. Other variables tested proved to be statistically insignificant.

## DISCUSSION

The use of RAI in thyroid disease therapy is well established due to its efficacy and simplicity, and is well tolerated although with some recorded side effects that are relatively less severe than other treatment modalities and that can be prevented or minimized if appropriate measures are taken (24). These include nausea and vomiting, and less commonly, radiation thyroiditis, gastritis and sialadenitis, the latter usually involving the parotid glands (25,26). The development of exacerbation of hyperthyroidism and hypersensitivity to RAIT are considered extremely rare (27,28). Iatrogenic hypothyroidism as a side effect is an expected outcome. In our hospital setting, due to economic constraints, most patients advocate for earlier hypothyroidism, and thus avoid the possibility of repeat RAIT.

The side effects of RAIT are found to be significantly dose-dependent or deterministic, hence the increasing severity of side effects proportional to the quantity of RAI received. Thus, ARs are more common in patients with WDTC and EUG than those with TG, and significantly more common with RAI for malignant than benign goiter. Most studies reporting ARs from RAIT involve patients with WDTC. We hope to do likewise when we have enough number of patients to achieve statistical significance.

The most common AR observed in our study was nausea and vomiting, consistent with the range of 50-67% cited in the literature (29). Other authors have described vomiting as being less common than nausea (30).

The frequency of radiation thyroiditis in our study, 27.5%, has been attributed to the large proportion of patients presenting for RAIT with substantial amount of functioning native thyroid tissue. This figure is higher than the predicted 1-5% range for patients treated with residual thyroid tissue (31). It has been frequently observed that both thyroid lobes are seen on radioiodine scans post-total thyroidectomy. In addition, dysphagia and dyspnea were observed after RAIT in these patients with significant residual thyroid tissue.

Radiation sialadenitis was not as common in our study, whereas it had been described as the most common side effect of RAIT in 11.5-67% of patients with WDTC treated with RAI (32,33,34). Despite the relatively low rate of ARs experienced overall, our goal would be no AR, as reported by Silberstein from 13200 sessions (17). Dysgeusia and xerostomia have been attributed to sialadenitis. Dysgeusia is caused by radioactive impairment of the taste buds leading to a change in the perception of taste (30).

The DoTS method classifies ARs as dose-related (augmented), non-dose-related (bizarre), dose-related and time-related (chronic), time-related (delayed), withdrawal (end of use), and failure of therapy (failure). Those RAIT related ARs described herein are dose-and time-related (13). The dose here would actually refer to the quantity of radioactivity administered, and not to the quantity of drug (iodide). Thus radioactivity-related side effects would be deterministic in nature (35,36). It is expected that these dose-related events will occur more commonly in patients treated with relatively higher therapy doses of 131I as for EUG and WDTC than those with TG. This held true for WDTC. However, TG patients had more reactions than EUG patients; this may be due to the fact that TG patients had more avid uptake of RAI than those with EUG, and thus a longer thyroid residence time.

The female preponderance of ARs was attributed to the pre-existing bias in the evaluated patients. Nevertheless, it has previously been noted that female patients were more likely to develop adverse drug reactions (37). The possible etiologies suggested include differences in cytochrome enzymes, hepatic and renal drug metabolism, body mass, as well as hormonal and immunologic factors (38,39). In all, women are 50-75% more likely to experience an AR than men (37).

### Study Limitations

The main limitations experienced were as follows: Routine neck ultrasonography to determine gland size was not performed on all patients. Thus, correlation between anatomical size and frequency of ARs could not be performed. Also, there is no formal system in place for reporting ARs from the use of radioisotopes and radiopharmaceuticals regionally or nationally. Protocols and standard operating procedures addressing the prevention and the management of these ARs should be instituted both nationally and for the West African region. In addition, ARs experienced after patient discharge might not have been reported promptly at the exact time of their occurrence. In instances when ARs are self-limiting, patients might forget to report them (36).

## CONCLUSION

RAIT remains a safe option for the treatment of benign and TG. ARs experienced are mainly mild to moderate in severity and mostly short-lived. The incidence of 0.8% in this study compares favorably with global figures. As larger doses of RAI for WDTC and TG were more commonly associated with ARs, our study suggests that these patients merit stronger prophylactic measures as well as closer monitoring for earlier detection and management of these reactions. Moreover, nuclear physicians administering RAIT should be prepared to treat adverse events should they arise despite preventive measures.

## Ethics

Ethics Committee Approval: Waived (retrospective study), Informed Consent: Not obtained (retrospective study).

Peer-review: External and internal peer-reviewed.

Financial Disclosure: The authors declared that this study has received no financial support.

## Figures and Tables

**Table 1:**
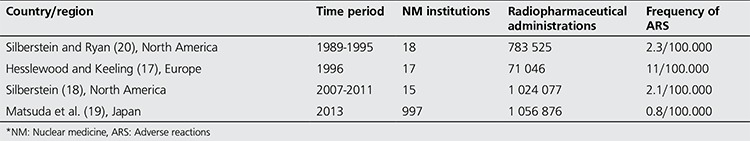
Previously reported frequency of adverse reactions to radiopharmaceuticals

**Table 2:**
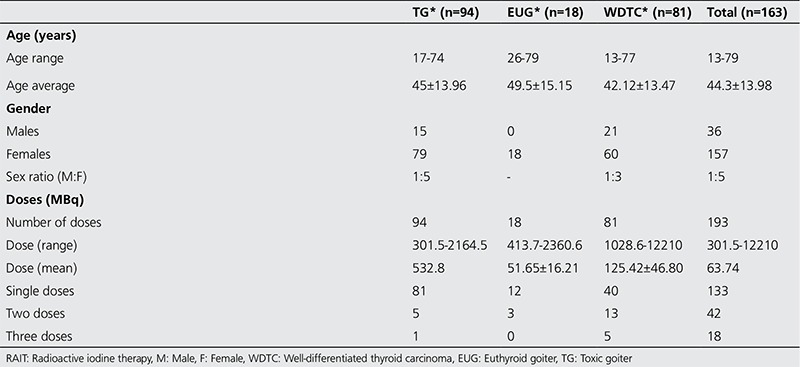
Characteristics of patients treated with radioactive iodine therapy between August 2006-June 2015

**Table 3:**
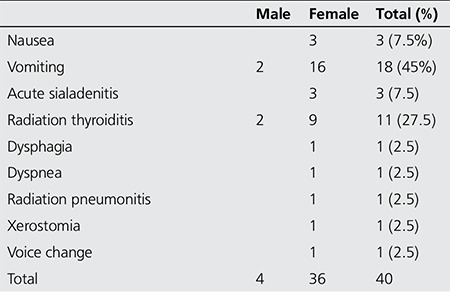
Adverse reactions experienced from radioactive iodine therapy displayed by gender

**Table 4:**
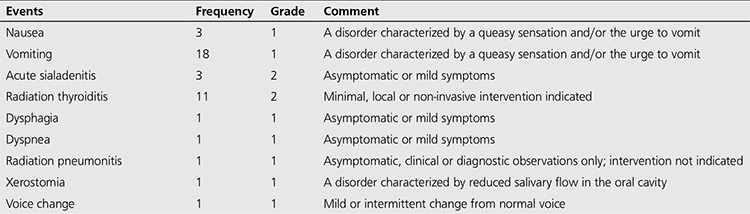
Grading of adverse reactions from radioactive iodine therapy (23)
